# PROTOCOL: Effectiveness of Interventions for the Prevention and Treatment of Obesity in Children and Adolescents From Low‐ and Middle‐Income Countries: A Systematic Review and Meta‐Analysis

**DOI:** 10.1002/cl2.70081

**Published:** 2025-12-16

**Authors:** Gerardo A. Zavala, Muhammad Asim, Aliya Ayub, Abdul Momin Rizwan Ahmad, Asha Prasad Pillai, Elena Pavlou, Avantika Sharma, Bilal Ahmad Khan, Hira Shakoor, Liina Mansukoski, Olga P. Garcia Obregon, Umber Khan, Zala Khan, Aliya Rehman, Badur Un Nisa, Humaira Bibi, Romania Iqbal, Suneel Gill, Urooj Ashfaq, Farman Ullah Khan, Zulfiqar Bhutta, Sarah Forberger, Kamran Siddiqi, Marc Suhrcke, Simon Walker, Zainab Samad, Saima Afaq

**Affiliations:** ^1^ Department of Health Sciences University of York York UK; ^2^ Institute of Public Health and Social Sciences Khyber Medical University Peshawar Pakistan; ^3^ Department of Human Nutrition & Dietetics, NUST School of Health Sciences National University of Sciences & Technology (NUST) Islamabad Pakistan; ^4^ Office of Research, Innovation and Commercialization Khyber Medical University Peshawar Pakistan; ^5^ Hull York Medical School University of York York UK; ^6^ Faculty of Natural Sciences Universidad Autonoma de Queretaro Santiago de Querétaro Mexico; ^7^ The Aga Khan University Karachi Pakistan; ^8^ Department of Rehabilitation Sciences Shifa Tameer e Millat University Islamabad Pakistan; ^9^ Institute of Psychiatry (IoP), Benazir Bhutto Hospital Rawalpindi Medical University Rawalpindi Pakistan; ^10^ Leibniz‐Insitute for Prevention Research and Epidemiology Bremen Germany; ^11^ Centre for Health Economics University of York York UK

## Abstract

Childhood obesity represents a major and growing public health challenge, with a disproportionate burden in low‐ and middle‐income countries (LMICs). This systematic review will address a critical evidence gap by synthesising existing research on the effectiveness of interventions for the prevention and treatment of childhood obesity in LMICs. By focusing exclusively on interventions implemented within LMIC contexts, the review will account for the unique socio‐cultural, economic, and environmental determinants that influence intervention delivery and effectiveness in these settings. A literature search will be conducted across MEDLINE/PubMed, Embase, CINAHL, and The Cochrane Library, without restrictions on publication date or language. Randomised controlled trials (RCTs) examining interventions for the prevention or treatment of overweight and obesity among children aged 5–9 years and adolescents aged 10–19 years will be included. The primary outcomes will be age‐adjusted body mass index (BMI), other measures of adiposity, and the prevalence of overweight and obesity. Secondary outcomes including dietary intake, physical activity, health‐related quality of life, and adverse events will be reported narratively but excluded from the meta‐analysis. Two independent reviewers will screen the studies, data extraction, and risk of bias assessment. Intervention effectiveness will first be summarised descriptively according to intervention type and key characteristics. Where appropriate, pooled effect sizes will be estimated using a random‐effects meta‐analysis. To explore and manage heterogeneity, analyses will be stratified by age group (children vs adolescents) and intervention purpose (prevention vs treatment). This review will identify effective strategies for preventing and treating childhood obesity in LMICs and explore the intervention features associated with successful outcomes. The findings will inform policy development and support the design and implementation of contextually appropriate interventions, contributing to global efforts to reduce obesity and prevent non‐communicable diseases (NCDs).

## 1 Background

### 1.1 Problem, Condition, or Issue

Obesity in children (5–11 years) and adolescents (5–19 years) is a critical public health challenge globally. Since the 1970s, the prevalence of childhood overweight and obesity has nearly tripled worldwide, contributing to 4 million annual deaths (Popkin 2020). The trends are particularly steep in low‐ and middle‐income countries (LMICs) (Pulungan et al. 2024). Going from a prevalence of 5% in 1980 to 15% in 2020 (Horta et al. 2022; NCD Risk Factor Collaboration (NCD‐RisC) 2024; Pulungan et al. 2024). In Asia, the combined prevalence of children and adolescent overweight and obesity is 23.2% (Mazidi et al. 2018), with similar prevalence in Latin America (20%) and some parts of Africa (between 11% and 13%) (Ayele et al. 2022; Corvalán et al. 2017; NCD Risk Factor Collaboration (NCD‐RisC) 2024).

Children and adolescents with obesity have a high likelihood (63%–67%) of becoming adults with obesity, which increases two to threefold the risk of developing non‐communicable diseases (NCDs), including cardiovascular diseases, type 2 diabetes, and cancer (Heidari‐Beni 2019; NCD Risk Factor Collaboration (NCD‐RisC) 2024). In LMICs, inadequate infrastructure and low household income make childhood obesity an even greater individual and societal challenge (Ling et al. 2023). A study across 56 LMICs found that childhood overweight and obesity contributed $237.55 per capita to the rise in annual medical costs. The expenses of non‐hospital healthcare, outpatient visits, medicine, and hospitalisation increased by $56.52, $14.27, $46.38, and $1975.06 per capita due to overweight and obesity, respectively (Ling et al. 2023).

Despite the growing burden of children and adolescent obesity in LMICs, there is scarce evidence on effective interventions in these resource‐constrained settings (Salam et al. 2020; Verstraeten et al. 2012). Existing systematic reviews predominantly focus on high‐income countries, overlooking interventions tailored to the unique socio‐cultural, economic, and environmental contexts of LMICs (Birch et al. 2019). A comprehensive review by Salam et al., including experimental and quasi‐experimental studies, found 654 studies overall; of these, only 83 were performed in LMICs, of which only 21 are randomised control trials (RCTs) (Salam et al. 2020).

### 1.2 The Intervention

**Figure 1:**
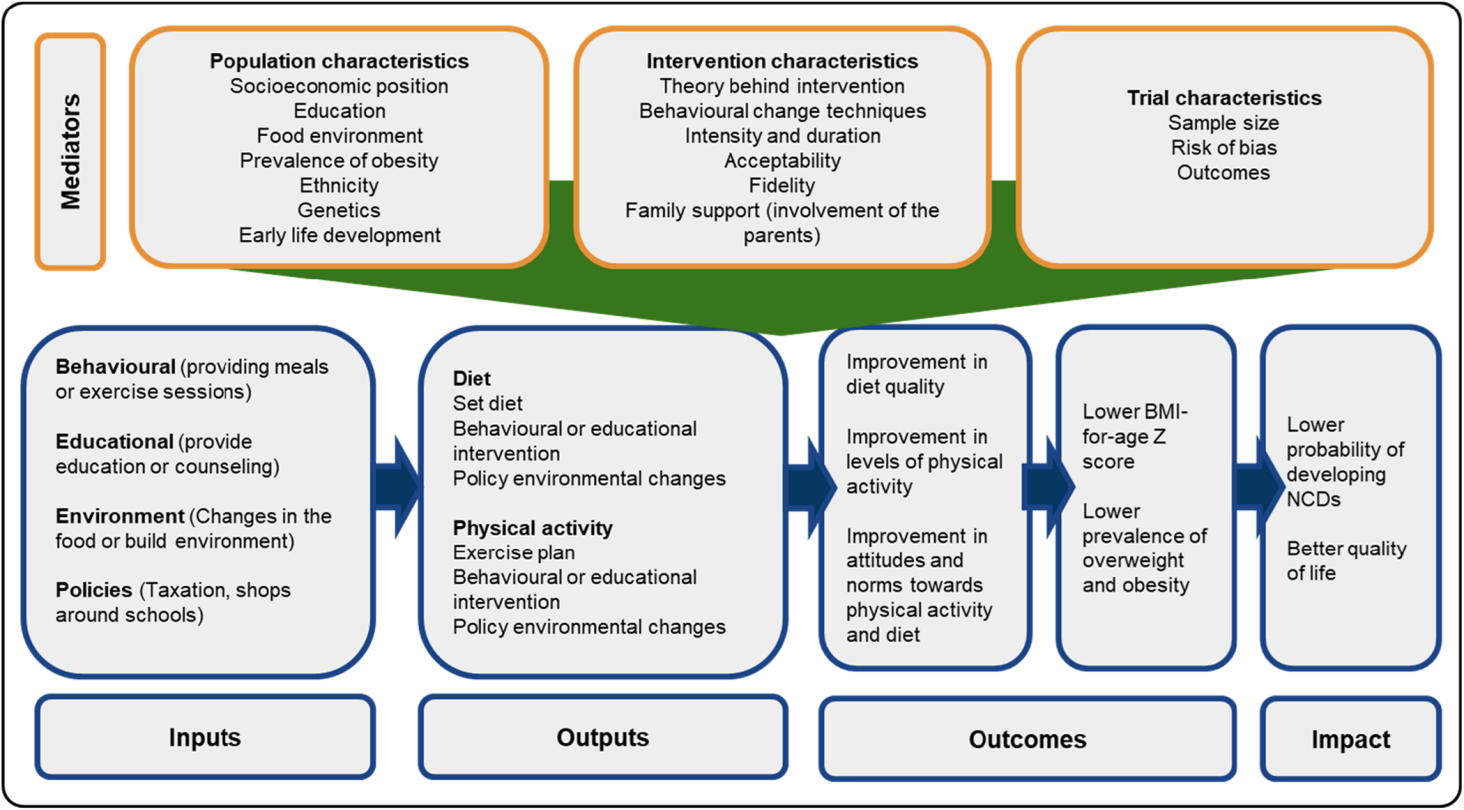
Logic model of change.

Interventions for addressing childhood (5–19 years)obesity in LMICs have been defined and structured comprehensively based on a logic model developed by our team Figure 1, drawing upon insights from global reviews to ensure adaptability across various contexts (Salam et al. 2020; Verstraeten et al. 2012). We categorised interventions into four main types: behavioural, educational, environmental, and pharmacological. Behavioural interventions focus on modifying diet and physical activity behaviours, including nutritional education to promote healthy eating habits, physical activity programmes to increase energy expenditure, and behaviour change techniques such as goal setting and self‐monitoring. Educational interventions aim to increase knowledge and awareness about obesity and its health risks, with programmes implemented in schools, communities, and healthcare facilities, including curriculum‐based programmes in schools and community workshops for parents and caregivers. Environmental interventions seek to create supportive settings that facilitate healthy choices and reduce obesogenic influences. These may include changes to the built environment—such as improving access to safe recreational spaces and healthy food outlets—as well as policy‐level changes like regulating the marketing of unhealthy foods to children, implementing school food standards, or introducing taxes on sugar‐sweetened beverages. Interventions can also involve modifying food availability and portion sizes in schools, promoting active transport (e.g., walking or cycling to school), and redesigning neighbourhoods to encourage physical activity and limit sedentary behaviours. Pharmacological interventions, though less commonly used in paediatric populations, involve the use of medications or supplements to aid in weight management, such as prescription weight loss drugs and nutritional supplements that promote weight loss or prevent weight gain.

### 1.3 How the Intervention Might Work

The logic model of change (Figure 1) outlines the pathways through which interventions aim to reduce calorie intake and increase energy expenditure, promoting healthy weight management among children and adolescents (Jarouliya and Keservani 2019). Grounded in established behavioural theories, this model incorporates insights from Jarouliya and Keservani (Jarouliya and Keservani 2019), who emphasise the importance of reducing caloric intake and increasing physical activity as primary mechanisms for weight management. The model also shows how behavioural change and weight management can be achieved through educational initiatives, behaviour change techniques, and environmental modifications (Deal et al. 2020). Interventions may be guided by specific behavioural theories such as the Social Cognitive Theory (Bagherniya et al. 2017), the Theory of Planned Behaviour (Stark et al. 2025), and the Health Belief Model (Nourian et al. 2016). However, many interventions in LMICs are developed pragmatically and may not be explicitly grounded in a single theoretical framework.

The successful delivery of interventions in LMICs requires careful consideration of the unique population, intervention, and trial characteristics inherent to these settings. For instance, educational initiatives increase knowledge and awareness about healthy behaviours, behaviour change techniques, self‐regulation and motivation (Michie et al. 2021), and environmental modifications create conducive settings for healthy living (Mazidi et al. 2018). By integrating these elements into the review process, the study aims to elucidate the factors influencing the effectiveness of interventions for the prevention and treatment of obesity in children and adolescents living in LMICs. The logic model thus connects the intervention components to intermediate and final outcomes, and each of the identified characteristics that might affect its effectiveness.

### 1.4 Why It Is Important to Do This Review

By narrowing the focus to LMICs, we aim to capture studies not included in previous global reviews and identify factors that make interventions effective in resource‐constrained settings, which will support intervention development and trial delivery. This effort is crucial for understanding not only what interventions ‘work’, but also to identify the trial, population, and contextual characteristics that influence their success (Goryakin et al. 2015). In LMICs, population characteristics such as poverty, food insecurity, limited parental education, and entrenched gender norms can create substantial barriers to healthy behaviours. Cultural beliefs around diet, physical activity, and body image may also affect how children and families engage with interventions. These factors can influence both the reach and impact of programmes, making interventions more or less effective depending on the local context (Barbosa Filho et al. 2016).

Intervention effectiveness is also shaped by design and delivery constraints common in LMICs. Limited infrastructure, lack of trained personnel, and weak regulatory environments can restrict the scope and sustainability of interventions. For example, children may attend school for fewer hours or irregularly, limiting exposure to school‐based programmes. In many settings, there are no formal regulations on food marketing or school meal standards. As a result, interventions may be shorter in duration, lower in intensity, and implemented under challenging conditions (Hawthorne et al. 2010; Zavala et al. 2022). Additionally, trials conducted in these contexts often face small sample sizes, limited follow‐up, and resource constraints, which can affect both the reliability of findings and their generalisability. These combined factors help explain why interventions may vary in effectiveness across LMICs (Bryant et al. 2014; Sedgwick 2015).

This review is needed to address several gaps and unresolved questions in the existing literature. In the latest Cochrane reviews (Spiga et al. 2024a, 2024b), out of 246, only 64 were delivered in LMICs. While subgroup analyses by country income level reported no statistically significant differences in effect sizes, these conclusions are constrained by both the small number of LMIC studies and the heterogeneity of contexts.

Moreover, many of the included trials were not primarily designed to assess adiposity‐related outcomes; BMI or BMI *z*‐scores were often secondary outcomes. This is a strength, since it includes a broad number of studies but also a limitation, as such trials may not have been adequately powered to detect meaningful changes in adiposity‐related outcomes, introducing the risk of underestimating or misestimating intervention effectiveness (Torgerson 2008).

In addition, our previous experience conducting reviews with LMIC‐specific search strategies has shown that a substantial number of relevant trials conducted in these settings are often missed by global reviews (Zavala et al. 2022). By focusing on LMICs, we aim to identify a broader and more representative body of evidence that reflects the diversity of interventions being tested in resource‐constrained settings. By examining the methodological limitations of trials and exploring context‐specific determinants of effectiveness, this review will contribute to providing evidence for the development and evaluation of more appropriate, scalable, and equitable interventions in LMICs.

In light of this underrepresentation, our review aims to address three key questions: (1) What is the effectiveness of interventions to prevent and treat childhood obesity in LMICs? (2) What are the key characteristics of these interventions? (3) How do these interventions vary in effect by age group, type of intervention, and setting within LMIC contexts? Thus, the objectives of this review are to examine the underrepresented contexts, assess the effectiveness of interventions for the prevention and treatment of childhood and adolescent overweight and obesity within LMIC contexts, and estimate the extent of heterogeneity across intervention effects. Given the anticipated volume and complexity of studies on children and adolescent interventions, we will address them in separate reports.

## 2 Methods

To ensure the highest standards of quality and rigour in our systematic review, we will adhere to the Campbell Collaboration Author Guidelines, following the Methodological Expectations of Campbell Collaboration Intervention Reviews (MECCIR). Given the anticipated volume and heterogeneity of studies, we will produce two separate reports: one focused on interventions targeting children (ages 5–9) and another on adolescents (ages 10–19). Furthermore, within each report, findings will be systematically categorised and analysed separately for prevention and treatment strategies.

### 2.1 Types of Studies

This review will include RCTs, including individual‐level and clustered designs. Quasi‐experimental and observational study designs will be excluded. Our goal is to use the most robust study design available, the RCT, to rigorously explore what works in LMICs. All interventions reported in peer‐reviewed journals will be considered without language restrictions. Studies conducted in any settings, such as schools, communities, households, and healthcare facilities, will be eligible for inclusion. We will document and report the number of identified study protocols that lack published results.

To ensure the reliability and completeness of the data included in the review, we will exclude grey literature and conference abstracts. These types of publications often lack the detailed information required to assess the risk of bias or to extract a comprehensive set of outcomes and variables, and thus do not meet the methodological standards necessary for inclusion in our synthesis.

### 2.2 Types of Participants

We will include RCTs involving participants aged between 5 and 19 years or interventions targeting the whole family, provided they report outcomes independently for the children. We will separate the reports between younger children (ages 5–9) and adolescents (ages 10–19). Given the variation in how age is reported across studies, we will take a flexible approach. Where exact age categories are not available, we will use the average age reported or the study‐defined age groupings. If this information is also unavailable, we will apply a pragmatic classification: studies conducted primarily in primary school settings will be categorised as targeting children, while those conducted in secondary school settings will be treated as targeting adolescents. If a study includes both groups without disaggregated results, it will be analysed as a mixed‐age group. In cases where studies provide subgroup analyses by age, these estimates will be disaggregated and entered as independent entries for children and adolescents, respectively. We will include studies involving children or adolescents with pre‐existing health conditions, including those conducted in hospital or clinical settings. Where available, these will be treated as a distinct subgroup within the narrative synthesis to explore potential differences in intervention effectiveness and characteristics.

### 2.3 Types of Interventions

#### 2.3.1 Interventions

The review will include any interventional study aimed at prevention and treatment of overweight and obesity such as behavioural modifications, which focus on improving diet and physical activity; educational programmes designed to enhance knowledge and skills around obesity prevention and management; environmental strategies will also be examined, which seek to create supportive surroundings that promote nutritional eating and active living (e.g., changes in the school canteen options, access to recreation facilities and availability of drinking fountains); In addition to these approaches, treatment‐specific interventions will also include pharmacological interventions involving the use of approved medications or nutritional supplements aimed at promoting weight loss or preventing weight gain, typically reserved for children with severe obesity or associated comorbidities and administered under medical supervision. Surgical interventions, such as bariatric procedures, will also be considered where applicable. Interventions delivered either as standalone approaches or as part of multi‐component programmes will be eligible for inclusion. We will not exclude studies based on the duration of the intervention.

#### 2.3.2 Comparator

Comparators will include true control (placebo or no intervention), usual care (defined by the study author), or alternative concomitant therapy, provided it was delivered in both the intervention and comparator arms.

### 2.4 Types of Outcome Measure

#### 2.4.1 Primary Outcomes

The primary outcomes of this review will be a change in adiposity using any standardised measurement, including *z*BMI *z*‐score, BMI, body fatness measured by dual‐energy X‐ray absorptiometry, bioelectrical impedance analysis (BIA), and air displacement plethysmography (peapod); and a change in the prevalence of overweight and obesity, defined by the study author. Self‐reported measures of primary outcomes will be excluded from the review to ensure data reliability (Althubaiti 2016). To ensure that effect estimates reflect interventions explicitly designed to impact obesity, we will include only trials where adiposity was the primary outcome. Trials reporting adiposity as a secondary outcome will be excluded, as these trials are often not adequately powered or of sufficient duration to detect meaningful changes in weight‐related outcomes (Torgerson 2008).

#### 2.4.2 Secondary Outcomes

We will identify and list all secondary outcomes reported, including dietary intake, physical activity, costs, cost‐effectiveness, and health‐related quality of life. These outcomes will be presented descriptively. Adverse events, however, will be summarised narratively to provide additional contextual detail.

### 2.5 Duration and Follow‐Up

We will extract and analyse follow‐up data at short‐term (< 4 months), medium‐term (4–11 months), and long‐term (> 12 months) intervals. However, an overall analysis will also be conducted using the last available follow‐up (end‐point) as defined by each trial to assess the general effectiveness of the interventions.

### 2.6 Types of Settings

We will include studies focusing on children and adolescents living in LMICs, as categorised by the World Bank into low‐income, lower‐middle‐income, and upper‐middle‐income countries (World Bank Organisation 2023). Studies conducted in any settings, such as schools, communities, households, and healthcare facilities, will be eligible for inclusion.

### 2.7 Search Methods for Identification of Studies

#### 2.7.1 Electronic Searches

The literature search for this systematic review was conducted in the following databases and platforms: MEDLINE via PubMed, Embase via Elsevier, CINAHL via EBSCOhost, and the Cochrane Library via Wiley. These platforms were selected for their coverage of clinical and public health trials, particularly those relevant to obesity and adiposity outcomes in LMICs, and align with recommendations from the Campbell Collaboration Handbook for Intervention Reviews (*Campbell Collaboration 2014*). The searches were executed sequentially, concluding on 03 July 2023 for MEDLINE/PubMed, 06 July 2023 for Embase, 04 July 2023 for CINAHL, and 07 July 2023 for The Cochrane Library. To ensure the inclusion of the most current evidence, we will update and rerun all searches after the protocol has been published. Consistent with best practices, search terms used have been documented to ensure reproducibility and transparency in our review process and can be accessed in Appendix A. All databases will be searched from inception with no date restrictions to ensure comprehensive coverage, particularly given that obesity is a relatively recent public health concern in LMICs, with most trials emerging from the 1990s onward.

#### 2.7.2 Search Strategy

We developed our search strategy using a combination of Medical Subject Headings (MeSH) and free‐text keywords to maximise sensitivity and capture variations in terminology across databases. The search strategy was crafted to include search terms relevant to children and adolescents' overweight and obesity, LMICs, obesity or overweight and RCT. The search strategy is based on existing systematic review studies (Brown et al. 2019; Flynn et al. 2022; Jacob et al. 2021; Kobes et al. 2018; Ribeiro et al. 2023; Salam et al. 2020; Verstraeten et al. 2012), which were conducted around interventions to treat and prevent obesity among children and adolescents and with guidance from the Cochrane Highly Sensitive Search Strategy for identifying randomised trials in MEDLINE: sensitivity‐ and precision‐maximising version (2008 revision). Keywords for LMICs were adapted from the Cochrane Effective Practice and Organisation of Care (EPOC) filter (Cochrane Effective Practice and Organisation of Care 2022) and further corroborated with the World Bank Organisation Classification updated as of 01 July 2022, Appendix A.

#### 2.7.3 Searching Other Resources

We will search the references of all included studies and include any relevant trials in our review. If multiple publications arise from the same trial, we will collate them and treat them as a single study. Only the primary report will be used for data extraction, and such trials will not be counted as independent interventions.

### 2.8 Data Collection and Analysis

#### 2.8.1 Description of Methods Used in Primary Research

##### 2.8.1.1 Selection of Studies

Studies will be screened through a two‐step approach: titles and abstracts will be assessed first, followed by the full texts. This process will involve two independent researchers per study at both steps, and any disagreements will be resolved through a third independent researcher (Campbell Collaboration Systematic Reviews). During full‐text screening, all reasons for exclusion will be documented using Covidence (Covidence 2017). We will examine retraction statements and errata to assess potential study limitations or exclusions in our review or meta‐analysis.

In multi‐arm studies, we will include all intervention and control groups that meet our eligibility criteria. If any groups are excluded from the analysis, we will document it in the ‘Table of Characteristics of Included Studies’. Multi‐arm studies will be coded and analysed carefully to avoid arbitrary omission of relevant groups or double‐counting of participants.

### 2.9 Data Extraction and Management

Searches will be exported as RIS files and imported into Covidence for de‐duplication (Covidence 2017). Screening of titles and abstracts, as well as full‐text screening, will be conducted using Covidence software (Covidence 2017). If the full text of included studies is not openly available or if information is missing, we will contact the corresponding author to obtain the manuscript and relevant outcome data. Data extraction will be independently carried out by two co‐authors, with any disagreements resolved by a third, experienced co‐author. A pre‐designed and pilot‐tested data extraction form will be used to collect key information across several broad categories. These include study characteristics (such as author, year of publication, study title, design, setting, country, funding source, conflicts of interest, and risk of bias assessment), participant characteristics (including age, sex, ethnicity, study population, inclusion and exclusion criteria, indicators of socioeconomic position such as socioeconomic status or index of deprivation, sample size, and information on withdrawals or loss to follow‐up), and intervention details (covering type, aim, components, parental involvement, comparator, duration, frequency, mode of delivery, provider, provider training, treatment integrity, attendance, mediators, moderators, and any adverse events). Outcome data will include both primary and secondary outcomes, with details on the outcome name, measurement tools used, timing of assessments, and any reported summary statistics such as means and standard deviations at baseline, follow‐up points, and post‐intervention, as well as reported mean differences (MD). We will seek important unpublished data from study authors to address gaps in included study reports, enhancing the review's comprehensiveness and accuracy, especially for details on the risk of bias assessments, intervention specifics, outcomes, and subgroup analyses.

### 2.10 Assessment of Risk of Bias in Included Studies

Two independent reviewers will assess the risk of bias for each included study using the Cochrane risk of bias tool for randomised trials version 2.0 (RoB 2) (Higgins and Green 2008; Higgins et al. 2011). This tool evaluates the risk of bias at the outcome level across five domains: (1) bias arising from the randomisation process, (2) bias due to deviations from intended interventions, (3) bias due to missing outcome data, (4) bias in measurement of the outcome, and (5) bias in selection of the reported result. We will document the sources of information used to make judgments for each domain (Higgins and Green 2008; Higgins et al. 2011). We will document the sources of information utilised for each risk of bias domain during quality assessment. Any assessments based on assumptions from non‐publicly available documents will be disclosed.

The overall risk of bias for the studies will be determined by considering the risk across all domains. If any domain is judged as ‘high risk’, the study will be considered at high risk of bias overall. If all domains are judged as ‘low risk’, the study will be considered at low risk of bias overall. Studies with one or more domains assessed as ‘unclear risk’ or a mix of ‘low’ and ‘unclear’ risk without any ‘high risk’ domains will be considered at an unclear overall risk of bias (Higgins et al. 2011).

### 2.11 Measures of Treatment Effect

For the primary outcome measures of BMI *z*‐score, BMI and weight, the mean and standard deviation or median and interquartile range will be extracted. For studies that report medians and IQRs instead of means and standard deviations, we will apply established statistical methods to approximate means and standard deviations when appropriate (Luo et al. 2018). Dichotomous outcome data will be analysed using risk ratios (RRs) with 95% confidence intervals (CIs), while continuous outcome data will be analysed using MD with 95% CI.

### 2.12 Unit of Analysis Issues

We will include all relevant effect sizes reported within each study for adiposity‐related outcomes, consistent with Cochrane reviews on childhood and adolescent obesity prevention (Spiga et al. 2024a, 2024b). This includes measures such as BMI, *z*BMI, and body fat percentage. To account for the dependency of multiple effect sizes from the same study, we will use robust variance estimation, allowing us to retain all relevant information while avoiding bias due to selective reporting (Pustejovsky and Tipton 2022).

For studies reporting multiple follow‐up time points, we will prioritise the longest follow‐up (end‐point) for the overall analysis. However, to avoid bias associated with selective time‐point reporting and to better account for within‐study correlations, we will also analyse estimates at each time point separately. If sufficient data are available, we will apply a multilevel meta‐analytic model to account for the hierarchical structure of the data and improve the precision of the pooled estimates (Van den Noortgate et al. 2015).

We will consider the impact of clustering, matching, or other non‐standard design features in the included studies. Specifically, cluster‐randomised trials and cross‐over trials will be analysed using appropriate methods to avoid underestimating or overestimating study precision (Higgins and Green 2008). For cluster‐randomised trials, we will adjust for clustering using the intracluster correlation coefficient (ICC) to ensure that the study's precision is accurately estimated. If the ICC is not reported in a study, we will use the average ICC from similar studies included in the review to adjust the effect size, as recommended by the Cochrane Handbook (Higgins and Green 2008). For crossover trials, we will account for carry‐over effects and analyse the data using within‐participant comparisons to avoid bias. These adjustments will be applied using inverse variance methods in the meta‐analysis.

### 2.13 Criteria for Determination of Independent Findings

For multiple reports stemming from a single study, we will identify and consolidate all available sources to ensure that each study is represented only once in the analysis. This process will involve cross‐referencing author names, study locations, sample sizes, and intervention details to detect and eliminate duplicate reports. Additionally, when a study reports multiple conceptually similar outcomes, we will review the protocol or trial registration to determine the primary outcome—particularly the one on which the sample size calculation was based—and prioritise this in the analysis.

### 2.14 Dealing With Missing Data

In handling missing outcome data, we will employ a systematic approach to minimise potential biases and ensure the robustness of our findings. First, we will attempt to obtain or clarify missing data by contacting the corresponding authors of the studies in question. This includes reaching out to individuals or organisations that may hold the necessary information, such as unpublished data sets or additional study details. If efforts to retrieve the missing data are unsuccessful, we will transparently document the extent and nature of the missing data and assess its potential impact on the results. When required, we will follow Cochrane methods to estimate missing standard deviations from medians, interquartile ranges, or other summary statistics (Higgins and Green 2008). Where feasible, we will perform sensitivity analyses to explore the implications of the missing data on our overall conclusions.

### 2.15 Assessment of Heterogeneity

Given the expected diversity in the interventions, delivery methods, and population settings across the studies, we will adopt a random‐effects model for our meta‐analysis. We will visually inspect the data in a forest plot and quantify heterogeneity using the *I*² statistic (Deeks et al. 2019), and also report the estimate of between‐study variance using tau‐squared (*τ*²) (Bagherniya et al. 2017; Borenstein 2023).

### 2.16 Assessment of Reporting Biases

To ensure the robustness of our systematic review, we will assess reporting biases, which include: publication bias, the selective omission of findings, and an overlap analysis (with other similar reviews). Publication bias, where studies with nonsignificant findings are less likely to be published, and selective reporting of outcome variables or statistical results can significantly skew the overall findings of a review. To detect and address these biases, we will utilise several strategies. For publication bias, we will conduct funnel plot analyses, where applicable, and perform statistical tests such as Egger's test to evaluate asymmetry in the funnel plots (Egger et al. 1997), which may indicate the presence of bias. We will also assess the risk of selective reporting by comparing the outcomes reported in the published studies with those outlined in the study protocols or trial registries when available (Higgins and Green 2008).

Furthermore, to mitigate the risk of bias related to overlapping studies across reviews, we will utilise the Graphical Representation of Overlap for OVErviews (GROOVE) tool (Bracchiglione et al. 2022). While GROOVE is traditionally used in overviews of systematic reviews, its application in our systematic review is justified due to the existence of several recent global reviews (Spiga et al. 2024a; Salam et al. 2020) that partially include LMIC‐focused interventions but underrepresent relevant trials identified through more targeted LMIC‐specific strategies. By generating a matrix that highlights overlaps between our review and similar global reviews, and providing a quantitative assessment of unique studies captured by our review, we can address potential bias by ensuring that our review does not disproportionately rely on a narrow subset of studies and accurately reflects the broader evidence base. The GROOVE tool will facilitate the comparison of included studies across multiple reviews and calculate the number and proportion of studies unique to each review.

### 2.17 Data Synthesis

#### 2.17.1 Meta Analysis

Given the expected diversity in the interventions, delivery methods, and population settings across the studies, we will adopt a random‐effects model for our meta‐analysis. Dichotomous outcome data will be analysed using RRs with 95% CIs, while continuous outcome data will be analysed using MD. We will quantify the heterogeneity using the I² statistic, which will enable us to classify the degree of heterogeneity as either low (0%–40%), moderate (30%–60%), substantial (50%–90%), or considerable (75%–100%), enhancing our understanding of the interventions' variable effects (Deeks et al. 2019; Higgins and Green 2008).

To minimise selective reporting and enhance transparency in our analytical approach, we will pre‐specify all planned pairwise meta‐analyses. These will include for each available primary outcome (e.g., BMI, *z*BMI): (1) no intervention or usual care versus dietary interventions; (2) no intervention or usual care versus physical Activity intervention; (3) no intervention or usual care versus combined dietary and physical activity interventions; and if available, (4) no intervention or usual care versus pharmacological or surgical interventions. These comparisons will be stratified by age group (children vs. adolescents) in different reports, intervention aim (prevention vs. treatment), and setting (e.g., school, community, clinical). Statistical analysis will be performed with Stata 19 (*Stata Statistical Software*, College Station, TX: StataCorp LLC.).

### 2.18 Subgroup and Sensitivity Analysis

In each of the reports (i.e., children and adolescents), prevention and treatment strategies will be analysed separately. We will conduct subgroup analyses according to the different intervention types—namely diet, physical activity, and combined approaches—and their modes of delivery (behavioural, educational, environmental, policy‐based, or a combination of these factors), and the duration of the interventions into short‐term (< 6 months), medium term (6 to < 12 months), and long‐term (12 months or more) following the Cochrane Handbook guidelines (Higgins and Green 2008), as well as the settings in which they are implemented (schools, communities, households, hospitals). Additionally, we will examine the impact of different comparators (e.g., no intervention, standard care, or alternative interventions), outcomes (e.g., changes in BMI, *z*BMI, waist circumference, and other adiposity measures), and funding sources (private vs. public) to understand how these factors influence the effectiveness of obesity interventions.

#### 2.18.1 Sensitivity Analysis

To ensure the robustness of our findings, we will conduct several sensitivity analyses. First, we will perform an analysis excluding studies that are identified as having a high risk of bias to assess how their exclusion impacts the overall results. Next, we will conduct sensitivity analyses by excluding studies that use different outcome measures, such as growth charts other than the WHO standards, to evaluate whether the choice of outcome measure influences our conclusions. Additionally, we will exclude studies where the population extends beyond our inclusion criteria, such as those that include participants outside the specified age range (e.g., those studies where the majority of the sample are children, but also some adults were included), to ensure that our results are consistent and specific to the target population.

### 2.19 Treatment of Qualitative Research

Not applicable.

### 2.20 Summary of Findings and Table

We will include a Summary of Findings and assess the certainty of the evidence using the Grading of Recommendations, Assessment, Development, and Evaluations (GRADE) approach. The outcomes to be included will focus on continuous measures, specifically *z*BMI and BMI, along with 95% CIs. The GRADE approach will be applied to evaluate the quality of the evidence for each outcome, taking into account factors such as study limitations, consistency of results, precision, directness of evidence, and potential publication bias (Zeng et al. 2021).

## Author Contributions

The development of the protocol involved contributions from Gerardo A. Zavala, Olga P. Garcia Obregon, Romania Iqbal, Zulfiqar Bhutta, Saima Afaq and Kamran Siddiqi, with additional input from Sarah Forberger, Muhammad Asim and Aliya Ayub. The systematic review methods were carried out by Gerardo A. Zavala, Kamran Siddiqi, Muhammad Asim and Bilal Ahmad Khan. Statistical analysis will be performed by Gerardo A. Zavala, Saima Afaq, Muhammad Asim and Bilal Ahmad Khan. Information retrieval will be conducted by a collaborative team including Asha Prasad Pillai, Elena Pavlou, Aliya Ayub, Abdul Momin Rizwan Ahmad, Avantika Sharma, Bilal Ahmad Khan, Hira Shakoor, Umber Khan, Zala Khan, Aliya Rehman, Badur Un Nisa, Humaira Bibi, Romania Iqbal, Suneel Gill, Urooj Ashfaq and Farman Ullah Khan.

## Conflicts of Interest

The authors declare no conflicts of interest.

## Preliminary Timeframe

We have already conducted the initial searches and begun the screening process for the included studies. With a large and capable team in place, we are confident in our ability to meet the project deadlines efficiently. We plan to submit the full systematic reviews by December 2026, which is within the 2‐year timeframe following protocol approval.

## Plans for Updating This Review

We plan to update the review every 5 years to ensure that it includes the latest evidence and reflects the most recent advancements in the field. The responsibility for these updates will rest with our team, which has the necessary resources and expertise to carry out this study. Regular updates will help ensure that the review remains a valuable and up‐to‐date resource for By narrowing the focus to LMICs, makers, practitioners, and researchers.

## Supporting information

Appendix A.

## Data Availability

The authors have nothing to report.
