# Supplementary material for: PROTOCOL: Effectiveness of Interventions for the Prevention and Treatment of Obesity in Children and Adolescents From Low‐ and Middle‐Income Countries: A Systematic Review and Meta‐Analysis
Source: Campbell Syst Rev. 2025 Dec 16;21(4):e70081. doi: 10.1002/cl2.70081 (PMC12706647; doi:10.1002/cl2.70081)
Supplement: Supplementary file 1 — Appendix A. [file CL2-21-e70081-s001.docx]

# **Appendix A: Search Strategies**

## **A.1 Ovid MEDLINE(R),**

### **Date Run: ALL <1946 to July 03, 2023>**

| **No.** | **Search Keywords** | **Results** |
| --- | --- | --- |
|  | Child/ (1912953) | (1912953) |
|  | Child, Preschool/ (989564) | (989564) |
|  | child*.tw. (1623592) | (1623592) |
|  | schoolchild*.tw. (15677) | (15677) |
|  | (kid or kids).tw. (9996) | (9996) |
|  | (boy* or girl*).tw. (274442) | (274442) |
|  | Minors/ (2819) | (2819) |
|  | minors.tw. (5587) | (5587) |
|  | underage*.tw. (1493) | (1493) |
|  | under-age*.tw. (5553) | (5553) |
|  | juvenil*.tw. (94023) | (94023) |
|  | Adolescent/ (2215561) | (2215561) |
|  | Young, adults/ (1011229) | (1011229) |
|  | adolescen*.tw | (346476) |
|  | teen*.tw. | (35071) |
|  | Preteen*.tw. | (380) |
|  | youth*.tw. | (96669) |
|  | preadolescen*.tw. | (3667) |
|  | pre-adolescen*.tw. | (1429) |
|  | youngster*.tw. | (2768) |
|  | Puberty/ | (14332) |
|  | pubert*.tw. | (43013) |
|  | pubescen*.tw. | (2817) |
|  | prepube*.tw. | (17029) |
|  | pre-pube*.tw. | (2653) |
|  | Pediatrics/ | (57873) |
|  | pediatric*.tw. | (368741) |
|  | paediatric*.tw. | (78414) |
|  | peadiatric*.tw. | (49) |
|  | Schools/ | (51354) |
|  | school*.tw. | (341390) |
|  | student*.tw. | (359308) |
|  | freshm?n.tw. | (2797) |
|  | (young adj3 adults).tw. | (92367) |
|  | (young adj3 people).tw. | (37544) |
|  | (young adj3 person).tw. | (1484) |
|  | (young adj3 adult).tw. | (41585) |
|  | (young adj3 women).tw. | (35522) |
|  | (young adj3 men).tw. | (21878) |
|  | college.tw. | (137425) |
|  | (pre-school* or preschool).tw. | (34279) |
|  | 42  1 or 2 or 3 or 4 or 5 or 6 or 7 or 8 or 9 or 10 or 11 or 12 or 13 or 14 or 15 or 16 or 17 or 18 or 19 or 20 or 21 or 22 or 23 or 24 or 25 or 26 or 27 or 28 or 29 or 30 or 31 or 32 or 33 or 34 or 35 or 36 or 37 or 38 or 39 or 40 or 41 | (5019796) |
|  | 43  (afghan* or africa* or albania* or algeria* or angola* or antigua* or barbuda* or argentin* or armenia* or aruba* or azerbaijan* or bahrain* or bangladesh* or bengal* or bangal* or barbados* or barbadian* or bajan or bajans or belarus* or belorus* or byelarus* or byelorus* or belize* or benin* or dahomey or bhutan* or bolivia* or bosnia* or herzegovin* or botswan* or batswan* or bechuanaland* or brazil* or brasil* or bulgaria* or burkina* or burkinese* or upper volta* or  burundi* or urundi* or cabo verde* or cape verde* or cambodia* or kampuchea* or khmer* or cameroon* or cameroun* or ubangi shari* or chad* or chile* or china* or chinese or colombia* or comoro* or comore* or comorian* or mayotte* or congo* or zaire* or costa rica* or &quot;cote d&#39;ivoir*&quot; or &quot;cote d&#39; ivoir*&quot; or cote divoir* or cote d ivoir* or ivory coast* or ivorian* or cook islands or croatia* or cuba or cuban or cubans or &quot;cuba&#39;s&quot; or cyprus* or cypriot* or czech* or djibouti* or  french somaliland* or dominica* or ecuador* or egypt* or united arab republic* or el salvador* or salvadoran* or guinea* or equatoguinea* or eritrea* or estonia* or eswatini* or swaziland* or swazi* or swati* or ethiopia* or fiji* or gabon* or gabonese* or gabonaise* or gambia* or ((Georgia or georgian or georgians) not (atlanta or california or florida)) or abkhazia* or abchasia* or south ossetia* or ghana* or gibraltar* or greece* or greek* or grecian* or grenada* or grenadian* or guam* or guatemala* or guyana* or guiana* or guyanese* or haiti* or hispaniola* or hondura* or hungary* or hungarian* or india* or indonesia* or iran* or iraq* or isle of man* or jamaica* or jordan* or kazakh* or kenya* or karabati* or korea* or kosovo* or kosova* or kyrgyz* or kirgiz* or kirghiz* or laos or lao or laotian* or latvia* or lebanon* or lebanese* or lesotho* or lesothan* or lesothonian* or basutoland* or mosotho* or basotho* or liberia* or libya* or jamahiriya* or lithuania* or macedonia* or madagasca* or malagasy* or malawi* or nyasaland* or malaysia* or malay* federation or sabah or sarawak or maldives* or maldivian* or indian ocean or mali or malian* or &quot;mali&#39;s&quot; or malta or maltese* or &quot;malta&#39;s&quot; or micronesia* or marshallese* or kiribati* or marshall island* or nauru or nauran or nauruans or &quot;naurian&#39;s&quot; or mariana or marianas or palau or paluan* or tuvalu* or mauritania* or mauritan* or mauritius* or agalega islands or mexico* or mexican* or moldova* or moldovia* or mongol* or montenegr* or montserrat* or morocco* or moroccan* or ifni or mozambique* or mozambican* or myanmar* or burma* or burmese or namibia* or niue or nepal* or new caledonia* or netherlands antill* or nicaragua* or niger* or oman or omani or omanis or &quot;oman&#39;s&quot; or pakistan* or palestin* or gaza* or west bank* or panama* or paraguay* or peru or peruvian* or &quot;peru&#39;s&quot; or philippine* or philipine* or phillipine* or phillippine* or filipino* or filipina* or poland* or polish or pole or poles or portugal* or Portuguese or puerto ric* or romania* or russia* or ussr* or soviet* or rwanda* or rwandese or ruanda* or ruandese or saint helen* or st helen* or samoa* or navigator island* or pacific island* or polynesia* or &quot;sao tome and principe*&quot; or sao tomean* or santomean* or saudi arabia* or saudi or saudis or senegal* or serbia* or seychell* or sierra leone* or slovak* or sloven* or melanesia* or solomon island* or norfolk island* or somali* or sri lanka* or ceylon* or &quot;saint kitts and nevis*&quot; or &quot;st kitts and nevis*&quot; or kittian* or nevisian* or saint lucia* or st lucia* or saint vincent* or st vincent* or vincentian* or grenadine* or sudan* or surinam* or syria* or tajik* or tadjik* or tadzhik* or tanzania* or tanganyika* or thai* or timor leste* or east timor* or timorese* or togo or togoles* or&quot;togo&#39;s&quot; or tonga* or trinidad* or tobago* or tunisia* or tokelau or turkiy* or turkey* or turk or turks or turkish or turkmen* or uganda* or ukrain* or uruguay* or uzbek* or vanuatu* or new hebrides* or venezuela* or vietnam* or viet nam* or wallis futuna or yemen* or yugoslav* or zambia* or zimbabwe* or rhodesia* or arab* countr* or middle east* or global south or sahara* or subsahara* or magreb* or maghrib* or west indies* or caribbean* or central america* or latin america* or south america* or central asia* or north asia* or northern asia* or southeastern asia* or south eastern asia* or southeast asia* or south east asia* or west asia* or western asia* or east europe* or eastern europe* or developing countr* or developing nation* or developing population* or developing world or less developed countr* or less developed nation* or less developed world or lesser developed countr* or lesser developed nation* or lesser developed world or under developed countr* or under developed nation* or under developed world or underdeveloped countr* or underdeveloped nation* or underdeveloped world or middle income countr* or middle income nation* or middle income population* or low income countr* or low income nation* or low income population* or lower income countr* or lower income nation* or lower income population* or underserved countr* or underserved nation* or underserved population* or under served population* or under served nation* or under served population* or deprived countr* or deprived population* or high burden countr* or high burden nation* or countdown countr* or countdown nation* or poor countr* or poor nation* or poor population* or poor world or poorer countr* or poorer nation* or poorer population* or poorer world or developing econom* or less developed econom* or underdeveloped econom* or under developed econom* or middle income econom* or low income econom* or lower income econom* or low gdp or low gnp or low gross domestic or low gross national or lower gdp or lower gnp or lower gross domestic or lower gross national or lmic or lmics or third world or lami countr* or transitional countr* or emerging econom* or emerging nation*).ti,ab,hw,kf. | (3184013) |
|  | exp Obesity/ or exp overweight/ | (271540) |
|  | Weight Gain/ | (35562) |
|  | exp Weight Loss/ | (49212) |
|  | obes*.af. | (471481) |
|  | (weight gain or weight loss).af. | (202047) |
|  | (overweight or over weight or overeat* or over eat*).af. | (106462) |
|  | weight change*.af. | (13920) |
|  | body mass index/ or body mass index.af. or BMI*.af. | (370327) |
|  | 44 or 45 or 46 or 47 or 48 or 49 or 50 or 51 | (849420) |
|  | randomized controlled trial.pt. | (595844) |
|  | controlled clinical trial.pt. | (95352) |
|  | randomized.ab | (608905) |
|  | placebo.ab. | (239577) |
|  | clinical trials as topic.sh. | (201056) |
|  | randomly.ab. | (411669) |
|  | trial.ti. | (288296) |
|  | 53 or 54 or 55 or 56 or 57 or 58 or 59 | (1533085) |
|  | exp animals/ not humans.sh. | (5135938) |
|  | 60 not 61 | (1411033) |
|  | 42 and 43 and 52 and 62 | (4802) |

## **A 2: Embase**

### **Date Run: (1974 to July 06, 2023)**

| **S.NO** | **Search Keys** | **Results** |
| --- | --- | --- |
|  | juvenile/ or boy/ or girl/ or preschool child/ or school child/ or toddler/ | (1013869) |
|  | preschool child/ | (620733) |
|  | child*.tw. | (2095812) |
|  | schoolchild*.tw. | (18322) |
|  | (kid or kids).tw. | (15022) |
|  | (boy* or girl*).tw. | (369845) |
|  | &quot;minor (person)&quot;/ | (906) |
|  | minors.tw. | (6836) |
|  | underage*.tw. | (1920) |
|  | under-age*.tw. | (7669) |
|  | juvenil*.tw. | (115668) |
|  | adolescent/ or adolescence/ | (1786949) |
|  | &#39;young adult&#39;/ | (511988) |
|  | adolescen*.tw. | (460181) |
|  | teen*.tw. | (48901) |
|  | Preteen*.tw. | (473) |
|  | youth*.tw. | (120469) |
|  | preadolescen*.tw. | (4292) |
|  | pre-adolescen*.tw. | . (1881) |
|  | youngster*.tw. | (3839) |
|  | pubert*.tw. | (59231) |
|  | pubescen*.tw. | (3189) |
|  | prepube*.tw | . (21381) |
|  | pre-pube*.tw. | (4412) |
|  | Pediatrics/ | (94437) |
|  | pediatric*.tw. | (582684) |
|  | paediatric*.tw. | (140117) |
|  | peadiatric*.tw. | (246) |
|  | school/ | (71539) |
|  | school*.tw. | (420923) |
|  | student*.tw. | (467276) |
|  | freshm?n.tw. | (3408) |
|  | (young adj3 adults).tw. | (124766) |
|  | (young adj3 people).tw. | (52213) |
|  | (young adj3 person).tw. | (2518) |
|  | (young adj3 adult).tw. | (55112) |
|  | (young adj3 women).tw. | (48298) |
|  | (young adj3 men).tw. | (26934) |
|  | college.tw. | (266846) |
|  | (pre-school* or preschool).tw. | (40653) |
|  | 1 or 2 or 3 or 4 or 5 or 6 or 7 or 8 or 9 or 10 or 11 or 12 or 13 or 14 or 15 or 16 or 17 or 18 or 19 or 20 or 21 or 22 or 23 or 24 or 25 or 26 or 27 or 28 or 29 or 30 or 31 or 32 or 33 or 34 or 35 or 36 or 37 or 38 or 39 or 40 | (5092439) |
|  | (afghan* or africa* or albania* or algeria* or angola* or antigua* or barbuda* or argentin* or armenia* or aruba* or azerbaijan* or bahrain* or bangladesh* or bengal* or bangal* or barbados* or barbadian* or bajan or bajans or belarus* or belorus* or byelarus* or byelorus* or belize* or benin* or dahomey or bhutan* or bolivia* or bosnia* or herzegovin* or botswan* or batswan* or bechuanaland* or brazil* or brasil* or bulgaria* or burkina* or burkinese* or upper volta* or burundi* or urundi* or cabo verde* or cape verde* or cambodia* or kampuchea* or khmer* or cameroon* or cameroun* or ubangi shari* or chad* or chile* or china* or chinese or colombia* or comoro* or comore* or comorian* or mayotte* or congo* or zaire* or costa rica* or &quot;cote d&#39;ivoir*&quot; or &quot;cote d&#39; ivoir*&quot; or cote divoir* or cote d ivoir* or ivory coast* or ivorian* or cook islands or croatia* or cuba or cuban or cubans or &quot;cuba&#39;s&quot; or cyprus* or cypriot* or czech* or djibouti* or french somaliland* or dominica* or ecuador* or egypt* or united arab republic* or el salvador* or salvadoran* or guinea* or equatoguinea* or eritrea* or estonia* or eswatini* or swaziland* or swazi* or swati* or ethiopia* or fiji* or gabon* or gabonese* or gabonaise* or gambia* or ((georgia  or georgian or georgians) not (atlanta or california or florida)) or abkhazia* or abchasia* or south ossetia* or ghana* or gibraltar* or greece* or greek* or grecian* or grenada* or grenadian* or  guam* or guatemala* or guyana* or guiana* or guyanese* or haiti* or hispaniola* or hondura* or hungary* or hungarian* or india* or indonesia* or iran* or iraq* or isle of man* or jamaica* or jordan* or kazakh* or kenya* or karabati* or korea* or kosovo* or kosova* or kyrgyz* or kirgiz* or kirghiz* or laos or lao or laotian* or latvia* or lebanon* or lebanese* or lesotho* or lesothan* or  lesothonian* or basutoland* or mosotho* or basotho* or liberia* or libya* or jamahiriya* or lithuania* or macedonia* or madagasca* or malagasy* or malawi* or nyasaland* or malaysia* or malay* federation or sabah or sarawak or maldives* or maldivian* or indian ocean or mali or malian* or &quot;mali&#39;s&quot; or malta or maltese* or &quot;malta&#39;s&quot; or micronesia* or marshallese* or kiribati* or marshall island* or nauru or nauran or nauruans or &quot;naurian&#39;s&quot; or mariana or marianas or palau or  paluan* or tuvalu* or mauritania* or mauritan* or mauritius* or agalega islands or mexico* or mexican* or moldova* or moldovia* or mongol* or montenegr* or montserrat* or morocco* or  moroccan* or ifni or mozambique* or mozambican* or myanmar* or burma* or burmese or namibia* or niue or nepal* or new caledonia* or netherlands antill* or nicaragua* or niger* or  oman or omani or omanis or &quot;oman&#39;s&quot; or pakistan* or palestin* or gaza* or west bank* or panama* or paraguay* or peru or peruvian* or &quot;peru&#39;s&quot; or philippine* or philipine* or phillipine* or phillippine* or filipino* or filipina* or poland* or polish or pole or poles or portugal* or Portuguese or puerto ric* or romania* or russia* or ussr* or soviet* or rwanda* or rwandese or ruanda* or ruandese or saint helen* or st helen* or samoa* or navigator island* or pacific island* or polynesia* or &quot;sao tome and principe*&quot; or sao tomean* or santomean* or saudi arabia* or saudi or  saudis or senegal* or serbia* or seychell* or sierra leone* or slovak* or sloven* or melanesia* or solomon island* or norfolk island* or somali* or sri lanka* or ceylon* or &quot;saint kitts and nevis*&quot; or &quot;st kitts and nevis*&quot; or kittian* or nevisian* or saint lucia* or st lucia* or saint vincent* or st vincent*  or vincentian* or grenadine* or sudan* or surinam* or syria* or tajik* or tadjik* or tadzhik* or tanzania* or tanganyika* or thai* or timor leste* or east timor* or timorese* or togo or togoles* or  &quot;togo&#39;s&quot; or tonga* or trinidad* or tobago* or tunisia* or tokelau or turkiy* or turkey* or turk or turks  or turkish or turkmen* or uganda* or ukrain* or uruguay* or uzbek* or vanuatu* or new hebrides* or venezuela* or vietnam* or viet nam* or wallis futuna or yemen* or yugoslav* or zambia* or zimbabwe* or rhodesia* or arab* countr* or middle east* or global south or sahara* or subsahara* or magreb* or maghrib* or west indies* or caribbean* or central america* or latin america* or south america* or central asia* or north asia* or northern asia* or southeastern asia* or south eastern asia* or southeast asia* or south east asia* or west asia* or western asia* or east europe* or eastern europe* or developing countr* or developing nation* or developing population* or developing world or less developed countr* or less developed nation* or less developed world or lesser developed countr* or lesser developed nation* or lesser developed world or under developed countr* or under developed nation* or under developed world or underdeveloped countr* or underdeveloped nation* or underdeveloped world or middle income countr* or middle income nation* or middle income population* or low income countr* or low income nation* or low  income population* or lower income countr* or lower income nation* or lower income population* or underserved countr* or underserved nation* or underserved population* or under served  population* or under served nation* or under served population* or deprived countr* or deprived population* or high burden countr* or high burden nation* or countdown countr* or countdown nation* or poor countr* or poor nation* or poor population* or poor world or poorer countr* or poorer nation* or poorer population* or poorer world or developing econom* or less developed econom* or underdeveloped econom* or under developed econom* or middle income econom* or  low income econom* or lower income econom* or low gdp or low gnp or low gross domestic or low gross national or lower gdp or lower gnp or lower gross domestic or lower gross national or  lmic or lmics or third world or lami countr* or transitional countr* or emerging econom* or emerging nation*).ti,ab,hw,kf. | (3867672) |
|  | exp Obesity/ | (660232) |
|  | body weight gain/ or body weight loss/ or body weight change/ | (125833) |
|  | obes*.af. | (775464) |
|  | (weight gain or weight loss).af. | (361659) |
|  | (overweight or over weight or overeat* or over eat*).af. | (140959) |
|  | weight change*.af. | (30639) |
|  | body mass index/ or body mass index.af. or BMI*.af. [mp=title, abstract, heading word, drug trade name, original title, device manufacturer, drug manufacturer, device trade name, keyword  heading word, floating subheading word, candidate term word] | (781262) |
|  | 43 or 44 or 45 or 46 or 47 or 48 or 49 | (1558830) |
|  | (random$ or factorial$ or crossover$ or &#39;cross over$&#39; or cross-over$ or placebo$ or (doubl$ adj blind$) or (singl$ adj blind$) or assign$ or allocat$ or volunteer$).tw. | (2818352) |
|  | exp crossover procedure/ | (75687) |
|  | exp double blind procedure/ | (211806) |
|  | exp randomized controlled trial/ | (794205) |
|  | exp single blind procedure/ | (52396) |
|  | exp clinical trial/ | (1860210) |
|  | clinical trial.tw. | (286866) |
|  | randomization/ | (99579) |
|  | (random$ adj8 control$).tw. | (663196) |
|  | 51 or 52 or 53 or 54 or 55 or 56 or 57 or 58 or 59 | (3894568) |
|  | 41 and 42 and 50 and 60 | (12125) |
|  | (conference abstract$ or conference review or conference paper or conference proceeding).db,pt,su. | (5589827) |
|  | 61 not 62 | (9295) |

## **A 3: CINAHL**

### **Date Run: 4 July, 2023**

| **S.No** | **Search Keywords** | | | **Results** |
| --- | --- | --- | --- | --- |
| S1 | (MH "Child+") | | | 760,668 |
| S2 | TI child* OR AB child* | | | 603,261 |
| S3 | TI schoolchild* OR AB schoolchild* | | | 4,644 |
| S4 | TI kid OR AB kid OR TI kids OR AB kids | | | 11,467 |
| S5 | TI boy* OR AB boy* | | | 51,250 |
| S6 | TI girl* OR AB girl* | | | 53,189 |
| S7 | (MH "Minors (Legal)") | | | 822 |
| S8 | TI minors OR AB minors | | | 36,941 |
| S9 | TI underage* OR AB underage* | | | 1,022 |
| S10 | TI under-age* OR AB under-age* | | | 1,662 |
| S11 | TI juvenil* OR AB juvenil* | | | 12,617 |
| S12 | (MH "Adolescence+") | | | 606,119 |
| S13 | (MH "Young Adult") | | | 289,653 |
| S14 | TI adolescen* OR AB adolescen* | | | 172,145 |
| S15 | TI teen* OR AB teen* | | | 22,018 |
| S16 | TI preteen* OR AB preteen* | | | 249 |
| S17 | TI pre-teen* OR AB pre-teen* | | | 130 |
| S18 | TI youth* OR AB youth* | | | 65,266 |
| S19 | TI preadolescen* OR AB preadolescen* | | | 1,564 |
| S20 | TI pre-adolescen* OR AB pre-adolescen* | | | 563 |
| S21 | TI youngster* OR AB youngster* | | | 1,022 |
| S22 | (MH "Puberty+") | | | 4,876 |
| S23 | TI pubert* OR AB pubert* | | | 7,187 |
| S24 | TI pubescen* OR AB pubescen* | | | 338 |
| S25 | TI prepube* OR AB prepube* | | | 2,260 |
| S26 | TI pre-pube* OR AB pre-pube* | | | 439 |
| S27 | (MH "Pediatrics+") | | | 23,005 |
| S28 | TI pediatric* OR AB pediatric* | | | 144,732 |
| S29 | TI paediatric* OR AB paediatric* | | | 31,309 |
| S30 | TI peadiatric* OR AB peadiatric* | | | 24 |
| S31 | (MH "Schools+") | | | 84,943 |
| S32 | TI school* OR AB school* | | | 177,352 |
| S33 | TI student* OR AB student* | | | 216,885 |
| S34 | TI freshm?n OR AB freshm?n | | | 1,457 |
| S35 | TI young N3 adults OR AB young N3 adults | | | 46,838 |
| S36 | TI young N3 people OR AB young N3 people | | | 26,627 |
| S37 | TI young N3 person OR AB young N3 person | | | 2,282 |
| S38 | TI young N3 adult OR AB young N3 adult | | | 46,838 |
| S39 | TI young N3 women OR AB young N3 women | | | 17,008 |
| S40 | TI young N3 men OR AB young N3 men | | | 9,688 |
| S41 | TI college OR AB college | | | 84,422 |
| S42 | TI pre-school* OR AB pre-school* | | | 2,216 |
| S43 | TI preschool OR AB preschool | | | 13,591 |
| S44 | S1 OR S2 OR S3 OR S4 OR S5 OR S6 OR S7 OR S8 OR S9 OR S10 OR S11 OR S12 OR S13 OR S14 OR S15 OR S16 OR S17 OR S18 OR S19 OR S20 OR S21 OR S22 OR S23 OR S24 OR S25 OR S26 OR S27 OR S28 OR S29 OR S30 OR S31 OR S32 OR S33 OR S34 OR S35 OR S36 OR S37 OR S38 OR S39 OR S40 OR S41 OR S42 OR S43 | | | 1,840,379 |
| S45 | TI ( afghan* or africa* or albania* or algeria* or angola* or antigua* or barbuda* or argentin* or armenia* or aruba* or azerbaijan* or bahrain* or bangladesh* or bengal* or bangal* or barbados* or barbadian* or bajan or bajans or belarus* or belorus* or byelarus* or byelorus* or belize* or benin* or dahomey or bhutan* or bolivia* or bosnia* or herzegovin* or botswan* or batswan* or bechuanaland* or brazil* or brasil* or bulgaria* or burkina* or burkinese* or upper volta* or burundi* or urundi* or cabo verde* or cape verde* or cambodia* or kampuchea* or khmer* or cameroon* or cameroun* or ubangi shari* or chad* or chile* or china* or chinese or colombia* or comoro* or comore* or comorian* or mayotte* or congo* or zaire* or costa rica* or "cote d'ivoir*" or "cote d' ivoir*" or cote divoir* or cote d ivoir* or ivory coast* or ivorian* or cook islands or croatia* or cuba or cuban or cubans or "cuba's" or cyprus* or cypriot* or czech* or djibouti* or french somaliland* or dominica* or ecuador* or egypt* or united arab republic* or el salvador* or salvadoran* or guinea* or equatoguinea* or eritrea* or estonia* or eswatini* or swaziland* or swazi* or swati* or ethiopia* or ﬁji* or gabon* or gabonese* or gabonaise* or gambia* or ((georgia or georgian or georgians) not (atlanta or california or ﬂorida)) or abkhazia* or abchasia* or south ossetia* or ghana* or gibraltar* or greece* or greek* or grecian* or grenada* or grenadian* or guam* or guatemala* or guyana* or guiana* or guyanese* or haiti* or hispaniola* or hondura* or hungary* or hungarian* or india* or indonesia* or iran* or iraq* or isle of man* or jamaica* or jordan* or kazakh* or kenya* or karabati* or korea* or kosovo* or kosova* or kyrgyz* or kirgiz* or kirghiz* or laos or lao or laotian* or latvia* or lebanon* or lebanese* or lesotho* or lesothan* or lesothonian* or basutoland* or mosotho* or basotho* or liberia* or libya* or jamahiriya* or lithuania* or macedonia* or madagasca* or malagasy* or malawi* or nyasaland* or malaysia* or malay* federation or sabah or sarawak or maldives* or maldivian* or indian ocean or mali or malian* or "mali's" or malta or maltese* or "malta's" or micronesia* or marshallese* or kiribati* or marshall island* or nauru or nauran or nauruans or "naurian's" or mariana or marianas or palau or paluan* or tuvalu* or mauritania* or mauritan* or mauritius* or agalega islands or mexico* or mexican* or moldova* or moldovia* or mongol* or montenegr* or montserrat* or morocco* or moroccan* or ifni or mozambique* or mozambican* or myanmar* or burma* or burmese or namibia* or niue or nepal* or new caledonia* or netherlands antill* or nicaragua* or niger* or oman or omani or omanis or "oman's" or pakistan* or palestin* or gaza* or west bank* or panama* or paraguay* or peru or peruvian* or "peru's" or philippine* or philipine* or phillipine* or phillippine* or ﬁlipino* or ﬁlipina* or poland* or polish or pole or poles or portugal* or portuguese or puerto ric* or romania* or russia* or ussr* or soviet* or rwanda* or rwandese or ruanda* or ruandese or saint helen* or st helen* or samoa* or navigator island* or paciﬁc island* or polynesia* or "sao tome and principe*" or sao tomean* or santomean* or saudi arabia* or saudi or saudis or senegal* or serbia* or seychell* or sierra leone* or slovak* or sloven* or melanesia* or solomon island* or norfolk island* or somali* or sri lanka* or ceylon* or "saint kitts and nevis*" or "st kitts and nevis*" or kittian* or nevisian* or saint lucia* or st lucia* or saint vincent* or st vincent* or vincentian* or grenadine* or sudan* or surinam* or syria* or tajik* or tadjik* or tadzhik* or tanzania* or tanganyika* or thai* or timor leste* or east timor* or timorese* or togo or togoles* or "togo's" or tonga* or trinidad* or tobago* or tunisia* or tokelau or turkiy* or turkey* or turk or turks or turkish or turkmen* or uganda* or ukrain* or uruguay* or uzbek* or vanuatu* or new hebrides* or venezuela* or vietnam* or viet nam* or wallis futuna or yemen* or yugoslav* or zambia* or zimbabwe* or rhodesia* or arab* countr* or middle east* or global south or sahara* or subsahara* or magreb* or maghrib* or west indies* or caribbean* or central america* or latin america* or south america* or central asia* or north asia* or northern asia* or southeastern asia* or south eastern asia* or southeast asia* or south east asia* or west asia* or western asia* or east europe* or eastern europe* or developing countr* or developing nation* or developing population* or developing world or less developed countr* or less developed nation* or less developed world or lesser developed countr* or lesser developed nation* or lesser developed world or under developed countr* or under developed nation* or under developed world or underdeveloped countr* or underdeveloped nation* or underdeveloped world or middle income countr* or middle income nation* or middle income population* or low income countr* or low income nation* or low income population* or lower income countr* or lower income nation* or lower income population* or underserved countr* or underserved nation* or underserved population* or under served population* or under served nation* or under served population* or deprived countr* or deprived population* or high burden countr* or high burden nation* or countdown countr* or countdown nation* or poor countr* or poor nation* or poor population* or poor world or poorer countr* or poorer nation* or poorer population* or poorer world or developing econom* or less developed econom* or underdeveloped econom* or under developed econom* or middle income econom* or low income econom* or lower income econom* or low gdp or low gnp or low gross domestic or low gross national or lower gdp or lower gnp or lower gross domestic or lower gross national or lmic or lmics or third world or lami countr* or transitional countr* or emerging econom* or emerging nation* ) OR AB ( afghan* or africa* or albania* or algeria* or angola* or antigua* or barbuda* or argentin* or armenia* or aruba* or azerbaijan* or bahrain* or bangladesh* or bengal* or bangal* or barbados* or barbadian* or bajan or bajans or belarus* or belorus* or byelarus* or byelorus* or belize* or benin* or dahomey or bhutan* or bolivia* or bosnia* or herzegovin* or botswan* or batswan* or bechuanaland* or brazil* or brasil* or bulgaria* or burkina* or burkinese* or upper volta* or burundi* or urundi* or cabo verde* or cape verde* or cambodia* or kampuchea* or khmer* or cameroon* or cameroun* or ubangi shari* or chad* or chile* or china* or chinese or colombia* or comoro* or comore* or comorian* or mayotte* or congo* or zaire* or costa rica* or "cote d'ivoir*" or "cote d' ivoir*" or cote divoir* or cote d ivoir* or ivory coast* or ivorian* or cook islands or croatia* or cuba or cuban or cubans or "cuba's" or cyprus* or cypriot* or czech* or djibouti* or french somaliland* or dominica* or ecuador* or egypt* or united arab republic* or el salvador* or salvadoran* or guinea* or equatoguinea* or eritrea* or estonia* or eswatini* or swaziland* or swazi* or swati* or ethiopia* or ﬁji* or gabon* or gabonese* or gabonaise* or gambia* or ((georgia or georgian or georgians) not (atlanta or california or ﬂorida)) or abkhazia* or abchasia* or south ossetia* or ghana* or gibraltar* or greece* or greek* or grecian* or grenada* or grenadian* or guam* or guatemala* or guyana* or guiana* or guyanese* or haiti* or hispaniola* or hondura* or hungary* or hungarian* or india* or indonesia* or iran* or iraq* or isle of man* or jamaica* or jordan* or kazakh* or kenya* or karabati* or korea* or kosovo* or kosova* or kyrgyz* or kirgiz* or kirghiz* or laos or lao or laotian* or latvia* or lebanon* or lebanese* or lesotho* or lesothan* or lesothonian* or basutoland* or mosotho* or basotho* or liberia* or libya* or jamahiriya* or lithuania* or macedonia* or madagasca* or malagasy* or malawi* or nyasaland* or malaysia* or malay* federation or sabah or sarawak or maldives* or maldivian* or indian ocean or mali or malian* or "mali's" or malta or maltese* or "malta's" or micronesia* or marshallese* or kiribati* or marshall island* or nauru or nauran or nauruans or "naurian's" or mariana or marianas or palau or paluan* or tuvalu* or mauritania* or mauritan* or mauritius* or agalega islands or mexico* or mexican* or moldova* or moldovia* or mongol* or montenegr* or montserrat* or morocco* or moroccan* or ifni or mozambique* or mozambican* or myanmar* or burma* or burmese or namibia* or niue or nepal* or new caledonia* or netherlands antill* or nicaragua* or niger* or oman or omani or omanis or "oman's" or pakistan* or palestin* or gaza* or west bank* or panama* or paraguay* or peru or peruvian* or "peru's" or philippine* or philipine* or phillipine* or phillippine* or ﬁlipino* or ﬁlipina* or poland* or polish or pole or poles or portugal* or portuguese or puerto ric* or romania* or russia* or ussr* or soviet* or rwanda* or rwandese or ruanda* or ruandese or saint helen* or st helen* or samoa* or navigator island* or paciﬁc island* or polynesia* or "sao tome and principe*" or sao tomean* or santomean* or saudi arabia* or saudi or saudis or senegal* or serbia* or seychell* or sierra leone* or slovak* or sloven* or melanesia* or solomon island* or norfolk island* or somali* or sri lanka* or ceylon* or "saint kitts and nevis*" or "st kitts and nevis*" or kittian* or nevisian* or saint lucia* or st lucia* or saint vincent* or st vincent* or vincentian* or grenadine* or sudan* or surinam* or syria* or tajik* or tadjik* or tadzhik* or tanzania* or tanganyika* or thai* or timor leste* or east timor* or timorese* or togo or togoles* or "togo's" or tonga* or trinidad* or tobago* or tunisia* or tokelau or turkiy* or turkey* or turk or turks or turkish or turkmen* or uganda* or ukrain* or uruguay* or uzbek* or vanuatu* or new hebrides* or venezuela* or vietnam* or viet nam* or wallis futuna or yemen* or yugoslav* or zambia* or zimbabwe* or rhodesia* or arab* countr* or middle east* or global south or sahara* or subsahara* or magreb* or maghrib* or west indies* or caribbean* or central america* or latin america* or south america* or central asia* or north asia* or northern asia* or southeastern asia* or south eastern asia* or southeast asia* or south east asia* or west asia* or western asia* or east europe* or eastern europe* or developing countr* or developing nation* or developing population* or developing world or less developed countr* or less developed nation* or less developed world or lesser developed countr* or lesser developed nation* or lesser developed world or under developed countr* or under developed nation* or under developed world or underdeveloped countr* or underdeveloped nation* or underdeveloped world or middle income countr* or middle income nation* or middle income population* or low income countr* or low income nation* or low income population* or lower income countr* or lower income nation* or lower income population* or underserved countr* or underserved nation* or underserved population* or under served population* or under served nation* or under served population* or deprived countr* or deprived population* or high burden countr* or high burden nation* or countdown countr* or countdown nation* or poor countr* or poor nation* or poor population* or poor world or poorer countr* or poorer nation* or poorer population* or poorer world or developing econom* or less developed econom* or underdeveloped econom* or under developed econom* or middle income econom* or low income econom* or lower income econom* or low gdp or low gnp or low gross domestic or low gross national or lower gdp or lower gnp or lower gross domestic or lower gross national or lmic or lmics or third world or lami countr* or transitional countr* or emerging econom* or emerging nation* ) | | | 646,159 |
| S46 | (MH "Obesity+") | | | 115,133 |
| S47 | (MH "Weight Gain") OR (MH "Weight Cycling") | | | 13,783 |
| S48 | (MH "Weight Loss") | | | 24,929 |
| S49 | TI obes* OR AB obes* | | | 125,997 |
| S50 | TI weight gain OR AB weight gain | | | 20,088 |
| S51 | TI weight loss OR AB weight loss | | | 33,105 |
| S52 | TI overweight OR AB overweight | | | 38,827 |
| S53 | TI over weight OR AB over weight | | | 3,611 |
| S54 | | TI overeat* OR AB overeat* | 1,503 | |

## **A 4: Cochrane improved 2,**

### **Date Run: 07, July,2023 16:23:45**

| **S.No** | **Search Keys** | **Results** |
| --- | --- | --- |
|  | MeSH descriptor: [Child] explode all trees | 78113 |
|  | child*:ti,ab,kw | 191993 |
|  | schoolchild*:ti,ab,kw | 1701 |
|  | (kid or kids):ti,ab,kw | 1515 |
|  | (boy* or girl*):ti,ab,kw | 12517 |
|  | MeSH descriptor: [Minors] this term only | 11 |
|  | minors:ti,ab,kw | 329 |
|  | underage*:ti,ab,kw | 333 |
|  | under-age*:ti,ab,kw | 406 |
|  | juvenil*:ti,ab,kw | 4833 |
|  | MeSH descriptor: [Adolescent] explode all trees | 125559 |
|  | MeSH descriptor: [Young Adult] explode all trees | 84768 |
|  | adolescen*:ti,ab,kw | 159795 |
|  | teen*:ti,ab,kw | 3380 |
|  | Preteen*:ti,ab,kw | 68 |
|  | youth*:ti,ab,kw | 9835 |
|  | preadolescen*:ti,ab,kw | 488 |
|  | pre-adolescen*:ti,ab,kw | 142 |
|  | youngster*:ti,ab,kw | 187 |
|  | MeSH descriptor: [Puberty] this term only | 402 |
|  | pubert*:ti,ab,kw | 2084 |
|  | pubescen*:ti,ab,kw | 73 |
|  | prepube*:ti,ab,kw | 1308 |
|  | pre-pube*:ti,ab,kw | 311 |
|  | MeSH descriptor: [Pediatrics] this term only | 1071 |
|  | pediatric*:ti,ab,kw | 36948 |
|  | paediatric*:ti,ab,kw | 8411 |
|  | peadiatric*:ti,ab,kw | 26 |
|  | MeSH descriptor: [Schools] this term only | 3250 |
|  | MeSH descriptor: [Schools, Nursery] explode all trees | 43 |
|  | MeSH descriptor: [Universities] explode all trees | 1454 |
|  | school*:ti,ab,kw | 44044 |
|  | student*:ti,ab,kw | 46302 |
|  | freshm?n:ti,ab,kw | 263 |
|  | (young near/3 adults):ti,ab,kw | 9856 |
|  | (young near/3 people):ti,ab,kw | 3539 |
|  | (young near/3 person):ti,ab,kw | 677 |
|  | (young near/3 adult):ti,ab,kw | 100221 |
|  | (young near/3 women):ti,ab,kw | 4067 |
|  | (young near/3 men):ti,ab,kw | 3637 |
|  | college:ti,ab,kw | 22024 |
|  | (pre-school* or preschool):ti,ab,kw | 45501 |
|  | {or #1-#42} | 418046 |
|  | #44 (afghan* OR africa* OR albania* OR algeria* OR angola* OR antigua* OR barbuda* OR argentin* OR armenia* OR aruba* OR azerbaijan* OR bahrain*OR bangladesh* OR bengal* OR bangal* OR barbados* OR barbadian* OR bajan  OR bajans OR belarus* OR belorus* OR byelarus* OR byelorus* OR belize* OR benin* OR dahomey OR bhutan* OR bolivia* OR bosnia* OR herzegovin* OR botswan* OR batswan* OR bechuanaland OR brazil* OR brasil* OR bulgaria*  OR burkina* OR burkinese* OR upper-volta* OR burundi* OR urundi* OR cabo- verde* OR cape-verde* OR cambodia* OR kampuchea* OR khmer* OR cameroon* OR cameroun* OR ubangi-shari* OR chad* OR chile* OR china* OR chinese OR  colombia* OR comoro* OR comore* OR comorian* OR mayotte* OR congo* OR zaire* OR costa-rica* OR (cote* AND *ivoir*) OR ivory-coast* OR ivorian*  OR cook islands OR croatia* OR cuba* OR cyprus* OR cypriot* OR czech* OR djibouti* OR french-somaliland* OR dominica* OR ecuador* OR egypt* OR united-arab-republic* OR el-salvador* OR salvadoran* OR guinea* OR equatoguinea* OR eritrea* OR estonia* OR eswatini* OR swaziland* OR swazi* OR swati* OR ethiopia* OR fiji* OR gabon* OR gabonese* OR gabonaise* OR gambia* OR ((georgia OR georgian OR georgians) NOT (atlanta OR california OR florida)) OR abkhazia* OR abchasia* OR south ossetia* OR ghana* OR gibraltar* OR greece* OR greek* OR grecian* OR grenada* OR grenadian* OR guam* OR guatemala* OR guyana* OR guiana* OR guyanese* OR haiti* OR hispaniola* OR hondura* OR hungary* OR hungarian* OR india* OR indonesia* OR iran* OR iraq* OR isle-of-man* OR jamaica* OR jordan* OR kazakh* OR kenya* OR karabati* OR korea* OR kosovo* OR kosova* OR kyrgyz* OR kirgiz* OR kirghiz* OR laos OR lao OR laotian* OR latvia* OR lebanon* OR lebanese* OR lesotho* OR lesothan* OR lesothonian* OR basutoland* OR mosotho* OR basotho* OR liberia* OR libya* OR jamahiriya* OR lithuania* OR macedonia* OR madagasca* OR malagasy* OR malawi* OR nyasaland* OR malaysia* OR malay-federation OR malaya-federation OR malayan-federation OR sabah OR sarawak OR maldives* OR maldivian* OR indian-ocean* OR mali*  OR malta* OR maltese* OR micronesia* OR marshallese* OR kiribati* OR marshall-island* OR nauru OR nauran OR nauruans OR nauran* OR mariana OR marianas OR palau OR paluan* OR tuvalu* OR mauritania* OR mauritan* OR  mauritius* OR agalega islands OR mexico* OR mexican* OR moldova* OR moldovia* OR mongol* OR montenegr* OR montserrat* OR morocco* OR  moroccan* OR ifni OR mozambique* OR mozambican* OR myanmar* OR burma* OR  burmese OR namibia* OR niue OR nepal* OR new-caledonia* OR netherlands- antill* OR nicaragua* OR niger* OR oman* OR pakistan* OR palestin* OR gaza* OR west-bank* OR panama* OR paraguay* OR peru* OR philippine* OR  philipine* OR phillipine* OR phillippine* OR filipino* OR filipina* OR poland* OR polish OR pole OR poles OR portugal* OR portuguese OR puerto-  ric* OR romania* OR russia* OR ussr* OR soviet* OR rwanda* OR rwandese OR ruanda* OR ruandese OR saint helen* OR st helen* OR samoa* OR navigator- island* OR pacific-island* OR polynesia* OR sao-tome* OR santomean* OR  saudi-arabia* OR saudi OR saudis OR senegal* OR serbia* OR seychell* OR sierra-leone* OR slovak* OR sloven* OR melanesia* OR solomon-island* OR norfolk-island* OR somali* OR sri-lanka* OR ceylon* OR saint-kitts* OR  st-kitts* OR kittian* OR nevisian* OR saint-lucia* OR st-lucia* OR saint- vincent* OR st-vincent* OR vincentian* OR grenadine* OR sudan* OR  surinam* OR syria* OR tajik* OR tadjik* OR tadzhik* OR tanzania* OR tanganyika* OR thai* OR timor-leste* OR east-timor* OR timorese* OR togo* OR tonga* OR trinidad* OR tobago* OR tunisia* OR tokelau OR turkiy* OR turkey* OR turk OR turks OR turkish OR turkmen* OR uganda* OR ukrain* OR uruguay* OR uzbek* OR vanuatu* OR new-hebrides OR venezuela* OR vietnam*  OR viet-nam* OR wallis futuna OR yemen* OR yugoslav* OR zambia* OR zimbabwe* OR rhodesia* OR arab-countr* OR arabic-countr* OR middle-east* OR global-south OR sahara* OR subsahara* OR magreb* OR maghrib* OR west-  indies* OR caribbean* OR central-america* OR latin-america* OR south- america* OR central-asia* OR north-asia* OR northern-asia* OR  southeastern-asia* OR south-eastern-asia* OR southeast-asia* OR south- east-asia* OR west-asia* OR western-asia* OR east-europe* OR eastern- europe* OR developing-countr* OR developing-nation* OR developing- population* OR developing-world OR less-developed-countr* OR less-developed-nation* OR less-developed-world OR lesser-developed-countr* OR  lesser-developed-nation* OR lesser-developed-world OR under-developed-countr* OR under-developed-nation* OR under-developed-world OR  underdeveloped-countr* OR underdeveloped-nation* OR underdeveloped-world OR middle-income-countr* OR middle-income-nation* OR middle-income-population* OR low-income-countr* OR low-income-nation* OR low-income-population* OR lower-income-countr* OR lower-income-nation* OR lower-income-population* OR underserved-countr* OR underserved-nation* ORunderserved-population* OR under-served-population* OR under-served-nation* OR under-served-population* OR deprived-countr* OR deprived-population* OR high-burden-countr* OR high-burden-nation* OR countdown-countr* OR countdown-nation* OR poor-countr* OR poor-nation* OR poor-population* OR poor-world OR poorer-countr* OR poorer-nation* OR poorer-population* OR poorer-world OR developing-econom* OR less developed-  econom* OR underdeveloped-econom* OR under-developed-econom* OR middle-income-econom* OR low-income-econom* OR lower-income-econom* OR low-gdp OR low-gnp OR low-gross-domestic OR low-gross-national OR lower-gdp OR  lower-gnp OR lower-gross-domestic OR lower-gross-national OR lmic OR lmics OR third-world OR lami-countr* OR transitional-countr* OR emerging-econom* OR emerging-nation*):ti,ab,kw | 214984 |
|  | MeSH descriptor: [Obesity] explode all trees | 21262 |
|  | MeSH descriptor: [Overweight] this term only | 6756 |
|  | MeSH descriptor: [Weight Gain] this term only | 3151 |
|  | MeSH descriptor: [Weight Loss] this term only | 7728 |
|  | MeSH descriptor: [Weight Cycling] this term only | 0 |
|  | obes*:ti,ab,kw | 53512 |
|  | (weight-gain or weight-loss):ti,ab,kw | 37205 |
|  | (overweight or over-weight or overeat* or over-eat*):ti,ab,kw | 21274 |
|  | weight-change*:ti,ab,kw | 4770 |
|  | MeSH descriptor: [Body Mass Index] this term only | 12408 |
|  | body-mass-index:ti,ab,kw | 48036 |
|  | BMI*:ti,ab,kw | 55107 |
|  | {or #45-#56} | 129740 |
|  | MeSH descriptor: [Controlled Clinical Trial] explode all trees | 38477 |
|  | randomized-controlled-trial:ti,ab,kw | 617950 |
|  | controlled-clinical-trial:ti,ab,kw | 174123 |
|  | randomized:ti,ab | 961576 |
|  | placebo:ti,ab | 356284 |
|  | clinical-trials:ti,ab,kw | 134466 |
|  | randomly:ti,ab | 307706 |
|  | trial:ti,pt | 824907 |
|  | {or #58-#65} | 1621394 |
|  | #43 and #44 and #57 and # | 66 5288 |
